# Anti-breast Cancer Enhancement of a Polysaccharide From Spore of *Ganoderma lucidum* With Paclitaxel: Suppression on Tumor Metabolism With Gut Microbiota Reshaping

**DOI:** 10.3389/fmicb.2018.03099

**Published:** 2018-12-17

**Authors:** Jiyan Su, Dan Li, Qianjun Chen, Muxia Li, Lu Su, Ting Luo, Danling Liang, Guoxiao Lai, Ou Shuai, Chunwei Jiao, Qingping Wu, Yizhen Xie, Xinxin Zhou

**Affiliations:** ^1^State Key Laboratory of Applied Microbiology Southern China, Guangdong Provincial Key Laboratory of Microbial Culture Collection and Application, Guangdong Institute of Microbiology, Guangzhou, China; ^2^School of Pharmaceutical Science, Guangzhou University of Chinese Medicine, Guangzhou, China; ^3^Guangdong Yuewei Edible Fungi Technology Co. Ltd., Guangzhou, China; ^4^Department of Breast Disease, Guangdong Provincial Hospital of Chinese Medicine, Guangzhou University of Chinese Medicine, Guangzhou, China; ^5^School of Pharmacy and Chemistry, Dali University, Dali, China; ^6^Guangdong Laboratory Animals Monitoring Institute, Guangzhou, China; ^7^School of Pharmacy, Guangxi University of Chinese Medicine, Xining, China

**Keywords:** spore of *Ganoderma lucidum*, paclitaxel, tumor metabolism, immune checkpoints, gut microbiota

## Abstract

Increasing evidence highlights the cardinal role of gut microbiota in tumorigenesis and chemotherapy outcomes. Paclitaxel (PTX), although as a first-line chemotherapy reagent for breast cancer, still requires for improvement on its efficacy and safety due to drug resistance and adverse effects. The present work explored the enhancement of a polysaccharide derived from spore of *Ganoderma lucidum* (SGP) with PTX in a murine 4T1-breast cancer model. Results showed that the combination of PTX and SGP displayed an improved tumor control, in which mRNA expression of several Warburg effect-related proteins, i.e., glucose transporter 3 (*Glut3*), lactate dehydrogenase A (*Ldha*), and pyruvate dehydrogenase kinase (*Pdk*), and the metabolite profile of tumor was evidently altered. Flowcytometry analysis revealed that the combination treatment recovered the exhausted tumor infiltration lymphocytes (TILs) via inhibiting the expressions of immune checkpoints (PD-1 and Tim-3), while PTX alone evidently increased that of CTLA-4. 16S rRNA sequencing revealed a restoration by the combination treatment on gut microbiota dysbiosis induced by PTX, especially that *Bacteroides, Ruminococcus*, and other 5 genera were significantly enriched while the cancer-risk genera, *Desulfovibrio* and *Odoribacter*, were decreased. Moreover, spearman correlation analysis showed that abundance of *Ruminococcus* was significantly negative-associated with the amount of frucotose-6-phosphate within the tumor. Collectively, the present study suggests the clinical implication of SGP as an adjuvant candidate for PTX against breast cancer, which possibly relies on the regulation of tumor metabolism and gut microbiota.

## Introduction

Breast cancer, as one of the most threatening carcinoma, has taken the first place of cancer-related deaths in women population worldwide (Li et al., 2016; DeSantis et al., 2017). With the advances of experimental and clinical researches, strategies against breast cancer mainly include surgical resection, adjuvant chemotherapy, radiotherapy, and hormone therapy (PDQ Adult Treatment Editorial Board). Nevertheless, their efficacies remain unsatisfactory, not only due to the increasing drug resistance and adverse effects, but also because there are limitations in the application of certain strategies, especially that triple-negative breast cancer does not respond hormonal or trastuzumab-based therapies (Reddy, 2011). On the other hand, it is quite inspiring that several immunnotherapy reagents are undergoing clinical trials against breast cancer due to their success in other types of cancer (Katz and Alsharedi, 2017). However, there are still challenges ahead to widen its optimal selection of ideal candidates, in which the response efficacy is closely related to the gut microbiome (Gopalakrishnan et al., 2018; Routy et al., 2018b). Therefore, chemotherapies, mainly employing paclitaxel (PTX), are still the most common and cost-effective treatments for breast cancer control. But adverse effects of PTX, such as hypersensitivity reactions, myelosuppression, and peripheral neuropathy, still disturb most of patients (Gupta et al., 2014; Starobova and Vetter, 2017). Hence, continued hard work is required for update of the current regimens, and even a revolution, to improve the efficacy meanwhile eliminating the adverse effects.

Nowadays, gut microbiota has been recognized as a regulator of both host metabolism and immune response (Gill et al., 2006). In particular, increasing evidence highlights its cardinal role in tumorigenesis (Sivan et al., 2015; Wong et al., 2017) and that in the outcomes of chemotherapy and immunotherapy (Gopalakrishnan et al., 2018; Routy et al., 2018b). This is mainly attributed to their intrinsic capcities of drug metabolism and the influence on host metabolizing homeostasis (Haiser et al., 2013; Wilson and Nicholson, 2017). Moreover, gut microbiota is found to be able to interfere tumor, especially that it is necessary to reverse the Warburg effect by accumulating butyrate within colorectal adenocarcinomas (Donohoe et al., 2014). Warburg effect is indeed a shift from oxidative phosphorylation to aerobic glycolysis (Warburg et al., 1924), which represented the metabolic nature of tumor microenvironment (TME) to support tumor growth and to evade immune destruction. It is characterized by increased glucose uptake and accumulation of lactate, even under normoxic conditions, as well as the corresponding upregulation of transporters, glycolytic enzymes and the relative signaling pathway proteins (Warburg, 1956; Ward and Thompson, 2012). Therefore, pathways and activities involved in tumor metabolism have been considered as novel targets in cancer therapy. For instance, metformin, a classic antidiabetic agent for treating type 2 diabetes, has been undergoing phase 2/3 clinical trials as adjuvant reagent in several cancer types, attributing to a reduction on the expression of monocarboxylate transporter 4 (MCT4) in cancer-associated fibroblasts (Romero et al., 2015). Meanwhile, convincing evidence has revealed that metformin would significantly change the gut microbiota community as well as the gut metabolome (Forslund et al., 2015; Wu et al., 2017). However, the concrete correlations between microbiota and metabolism underlying these observations remain largely unknown.

*Ganoderma lucidum* (Leyss. et Fr.) Karst. is one of the most extensively studied basidiomycotina mushroom as a functional food and chemopreventive agent, especially in traditional Chinese medicine and other Asian folk medicine (Oliveira et al., 2014). Numerous documents have revealed that *G. lucidum* exerts anti-cancer effects not only via cancer cell-targeting approaches, such as cell cycle arrest (Wu et al., 2012), apoptosis induction (Dai et al., 2017), and migration inhibition (Tsao and Hsu, 2016), but also, more importantly, through ways of immune enhancement (Li et al., 2015; Sun et al., 2015). Recently, active components from the spore of *G. lucidum* (SG) have been unveiled versatile biological activities owing to the advance in sporoderm-breaking technology, especially the activities contributing to its anticancer potential (Wang et al., 2012; Na et al., 2017). In our previous study, it was found that a polysaccharide from SG (SGP) was able to potentiate the cytotoxicity T cell (Tc)-based tumor immune surveillance with a benefit reshaping on gut microbiota (Su et al., 2018). In the present study, the improvement potential of SGP on the antitumor activity of PTX was investigated from the perspective of tumor metabolism and gut microbiota.

## Materials and Methods

### Animals

Female Balb/c mice (6 to 8 week old, weighting 18–22 g) were provided by Guangdong Medical Laboratory Animal Center (Guangzhou, Guangdong, China). The mice were raised in specific pathogen-free condition (23 ± 2°C, 50 ± 5% humidity) in a 12 h light/dark cycle with food and water *ad libitum*. After 7-day acclimatization, the experiment was performed with the approval by Guang-dong Institute of Microbiology Laboratory Animal Ethics Committee according to the guidelines (permission number: GT-IACUC201708231).

### Preparation for Polysaccharide of the Sporoderm-Breaking Spore of *G. lucidum* (SGP)

SGP were prepared as described previously (Su et al., 2018). The sporoderm-breaking SG was provided by Guangdong Yuewei Edible Fungi Technology Co. Ltd. In brief, the spore was extracted with boiling distilled water. The extract was then concentrated, following by 2–3 cycles of precipitation with anhydrous ethanol (final percentage of ethanol was 85%), and dialysis. Finally, the 3.5–100 kDa dialysate was pooled, concentrated, and lyophilized, to obtain SGP with a yield of 0.4%. Polysaccharide content of SGP is about 50%, which is mainly made up of glucose with an average molecular weight (Mw) of 3.6 kDa as reported previously (Su et al., 2018).

### Cell Culture

Murine metastatic breast cancer 4T1 cell line was bought from Cell bank of Chinese Academy of Sciences, Shanghai, China. 4T1 cells were cultured in high glucose DMEM medium (4.5 mg/mL, Gibco, NY, USA) containing 10% fetal bovine serum (FBS, Gibco, NY, USA) and 1% penicillin/streptomycin (Gibco, NY, USA), and maintained in incubators at 37°C under an atmosphere of 5% CO_2_.

### 4T1-Breast Cancer Model Induction and Treatment

Murine 4T1-breast cancer model was established as described by Zhang et al. (2017) with mild modification. Briefly, 4T1 tumor cells were injected subcutaneously (*s.c*.) into the right forleg armpit of the subjected Balb/c mice (0.1 mL/mouse, 1 × 10^5^ cells/mouse). The tumor-bearing mice were randomly divided into Model group, PTX group (Hainan Quanxing Pharmaceutical Co. Ltd., Hainna, China) and the two combination treatment groups (SLP and SHP groups), 9 for each. Another 9 mice (as Normal group) were injected subcutaneously (*s.c*.) with 0.1 mL complete DMEM medium at the similar site. Over the following 21 days, PTX group was intraperitoneally injected (*i. p*.) with PTX at a dose of 12.5 mg/kg twice a week. The SLP group was administrated orally with SGP (200 mg/kg, *p.o*.) once a day, in addition to the twice-a-week intraperitoneal injection of PTX (12.5 mg/kg). The SHP group was administrated orally with SGP (400 mg/kg, *p.o*.) once a day, together with the PTX treatment (12.5 mg/kg). Normal group and Model group received equal volume of saline. In the following 21 days, tumor volume was measured with an electronic vernier caliper every 3 days since 6th day. The volume was calculated as *V* = *a* × *b*^2^/2, where *a* indicated the longer diameter, and *b* indicated the shorter diameter. On the 22th day, all animals were blooded from orbital plexus, and then sacrificed by cervical dislocation to harvest tumors. Tumors were weighted, photographed, segmented, and then stored according to different purposes immediately.

### Tumor Infiltrating Lymphocyte (TIL) Isolation and Flow Cytometry Analysis

Tumor segments kept in pre-cold PBS were used for TIL isolation and analysis. The segments were minced and digested in 3 mL digestive medium, which was mainly composed of basic RPMI160 medium supplemented with 0.1% Type IV collegenase (Invitrogen, Thermo Fisher Scientific, Grand Isle, NY, USA), 350 U/mL DNAse I (Roche, Basel, Switzerland), and 1% penicillin-streptomycin. Then they were ground in pre-cold PBS by passing through a 70 μm strainer, washed, and resuspended in basic RPMI160 medium. TILs from the obtained cell suspension were separated with Mouse 1× Lymphocyte Separation Medium (Dakewe Biotechnology Co. Ltd., Shenzhen, China) according to the manufacture' instruction. TILs were stained with FITC anti-mouse CD3 (2.5 μg/test), PE- Cyanine5 anti-mouse CD4 (0.0625 μg/test), APC-Cyanine7 anti-mouse CD8 (0.25 μg/test), PE anti-mouse CD 152 (cytotoxic T-lymphocyte-associated protein-4, CTLA-4, 0.25 μg/test), APC anti-mouse CD 273 (programmed cell death protein-1, PD-1, 1 μg/test), PE- Cyanine7 anti-mouse CD366 (T-cell immunoglobulin and mucin-domain containing-3, Tim-3, 0.25 μg/test), at 4°C in dark for 30 min. All the above antibodies were purchased from eBioscience, Thermo Fisher Scientific (Grand Isle, NY, USA). After two washes with pre-cold PBS, T cell subsets and the immune checkpoint expressions in TIL were enumerated with a FACS Canto II cytometer, and the data was analyzed by Diva software (version 6.1.3).

### Immunohistochemistry (IHC) for ki67

Immunohistochemistry for ki67 was performed with the formalin-fixed, paraffin-embedded tumor segment. Firstly, the slides were deparaffinized. Antigen retrieval was carried out by incubation in saline-sodium citrate buffer (pH 6.0) via autoclaving. After being washed with PBS, the sections were subjected to endogenous peroxidase blocking with 3% H_2_O_2_ in dark for 25 min. Then they were blocked with 3% bovine serum albumin (BSA), incubated with primary antibody against mouse ki67 (1:300, Servicebio, Wuhan, China) at 4°C overnight, following by the incubation with horse reddish peroxidase (HRP)-conjugated secondary antibodies at room temperature for 50 min. Lastly, the sections were stained with 3,3N-diaminobenzidine tertrahydrochloride substrate (DAB) and counterstained with hematoxylin. The mean density of positive area was calculated as ratio of integrated optical density (IOD) to the total pixel of each picture (IOD/10^6^ pixel), which was analyzed by Image Pro Plus 6.0 software (Media Cybernetics, Silver Spring, USA).

### Total RNA Extraction and Quantitative Real-Time PCR

One of the tumor segments was kept in sample protector for RNA/DNA (Takara Bio, Inc., Shiga, Japan) for quantitative real-time PCR (q-PCR). Total RNAs from tumors were extracted with TRIzol according to the manufacturer's instructions (Invitrogen, Thermo Fisher Scientific, Grand Isle, NY, USA). Two micrograms of total RNA was reverse transcribed using the ReverAid First Strand cDNA Synthesis Kit (Thermo Scientific, Inc., MA USA) following the supplier's protocol. The reactions were incubated at 25°C for 5 min, then at 42°C for 60 min, then at 70°C for 5 min, and the products were stored at −20°C before used. The PCR primer sequences are listed in Table 1. q-PCR reactions were conducted with SYBR® Premix Ex Taq™ II (Takara Bio, Inc., Shiga, Japan), in an StepOnePlus Real-Time PCR system (Thermo Fisher Scientific, Grand Isle, NY, USA). The program was as follows: a precycling stage at 95°C for 30 s, then 40 cycles of annealing at 95°C for 5 s, 60°C for 34 s. Fluorescence was measured at the end of each annealing step, and the melting curves were monitored to confirm the specificity of the PCR products. mRNA expression levels of target genes relative to control gene *Gapdh* was determined with the 2^−ΔΔ^ Ct method.

**Table 1:** Primers for quantitative real-time PCR.

**Gene name**	**GenBank accession**	**Primer**		**Product length (bp)**
*Glut1*	NM_011400.3	Sense	caa tgc tgt gtt cta cta ctc	252
		Antisense	gcc acg atg ctc aga tag	
*Hif1a*	NM_001313920.1	Sense	tga tgt gga tag cga gg	190
		Antisense	tgg cag tga tgg tag gtt	
*C-myc*	NM_001177352.1	Sense	gct ctg ctc tcc atc cta	120
		Antisense	agt aac tcg gtc atc atc tc	
*P53*	NM_001127233.1	Sense	tgg aag aca ggc aga ctt	151
		Antisense	gtg atg atg gta agg ata ggt	
*Glut3*	NM_011401.4	Sense	tcc agc cgc ttc tca tct cca t	148
		Antisense	gta ttg acc acg cct gct cca a	
*Ldha*	NM_001136069.2	Sense	gct gct gat cgt ctc caa tcc a	294
		Antisense	acc tcc ttc cac tgc tcc ttg t	
*Pdk*	NM_172665.5	Sense	gct acg gga cag atg cgg tta t	121
		Antisense	cag tcg tca gcc tcg tgg tt	
*Gapdh*	NM_008084.3	Sense	atg gtg aag gtc ggt gtg aac g	233
		Antisense	cgc tcc tgg aag atg gtg atg g	

### Metabolomics of Tumor Tissue

For metabolomics analysis, tumor segment was frozen in liquid nitrogen immediately after harvested and stored at −80°C until detection. The untargeted metabolomics profiling was performed by gas chromatograph–time-of-flight mass spectrum (GC-TOF/MS) on XploreMET platform (Metabo-Profile, Shanghai, China). The sample preparation procedure and GC-TOF/MS analysis were conducted as described previously with mild modification (Wang et al., 2013). More details are provided in the Table S1. The data result sets containing all the m/z value, retention time and ion peak area of each sample were exported to the multivariate statistical software SIMCA (version 14.0, Umetrics, Umea, Sweden) for the subsequent partial least squares discrimination analysis (PLS-DA). The differential metabolites between groups were obtained using a multi-criteria assessment in the orthogonal partial least square discrimination analysis (OPLS-DA) model, which combines the strength of both contribution (variable importance in projection, VIP) and variable reliability (correlation coefficients, Corr.). And then they were confirmed following by the univariate statistical analysis (student *T*-test) using the SPSS 22.0 software (IBM, USA). Finally, the differential metabolites that were responsible for the separation between two groups were selected and identified.

### 16S rRNA Gene Sequence Analysis of Gut Microbiota in Cecum

Caecum content was frozen in liquid nitrogen immediately after harvested and stored at −80°C until sequencing. Total DNA from the caecum content was extracted with Fast DNA SPIN extraction kits (MP Biomedicals, Santa Ana, CA, USA) following the manufacturer's recommendations. The bacterial 16S rRNA gene V3-4 region was amplified by PCR using the sense primer (5′-ACTCCTACGGGAGGCAGCA-3′) and the anti-sense primer (5′-GGACTACHVGGGTWTCTAAT-3′). PCR amplicons were purified with Agencourt AMPure Beads (Beckman Coulter, Indianapolis, IN) and quantified individually using the PicoGreen dsDNA Assay Kit (Invitrogen, Carlsbad, CA, USA). Next, amplicons were pooled in equal amounts, and pair-end 2 × 300 bp sequencing was performed using the Illlumina MiSeq platform with MiSeq Reagent Kit v3 (Zhao et al., 2013). High quality sequencing data and predicted function data were obtained by QIIME (Version 1.8.0), MOTHUR (version 1.31.2), and PICRUst (http://picrust.github.io/picrust/). The following statistics was performed by R software. α-diversity was evaluated by Ace, chao, simpson, and shannon index. β-diversity was assessed with the uniFrac distance-based principal coordinates analysis (PCoA) and analysis of similarities (ANOSIM). Taxon-based analysis and linear discriminant analysis with effect size (LEfSe) were applied to identify specific taxa microbes among groups using the default parameters (Segata et al., 2011). The predicted genes and their functions were aligned to Kyoto Encyclopedia of Genes and Genomes (KEGG) database, and differences among groups were compared through software STAMP4. Relationship between the “metabolite-bacterial genus” were performed by Spearman correlation analysis based on the abundance datasets.

### Statistics

Statistical analysis was performed with SPSS 22 (IBM Corp., NY, USA). Datasets from each experiment was subjected to normal distribution test firstly. If they followed normal distribution, one-way analysis of variance (ANOVA) was conducted following by different parametric test depending on test for homogeneity of variance, otherwise the data was compared by Kruskal–Wallis H-Test. In ANOVA, *post–hoc* LSD test was applied for difference analysis under homogeneity of variance, if not, a Dunnett's test would be applied. For tumor volume change comparison, repeat measurement ANOVA was performed with general linear model. For bioinformatics analysis, *p*-values were corrected (FDR < 0.05) to control multiple hypothesis testing errors.

## Results

### SGP Promoted the Anti-cancer Activity of PTX

**Figure 1:**
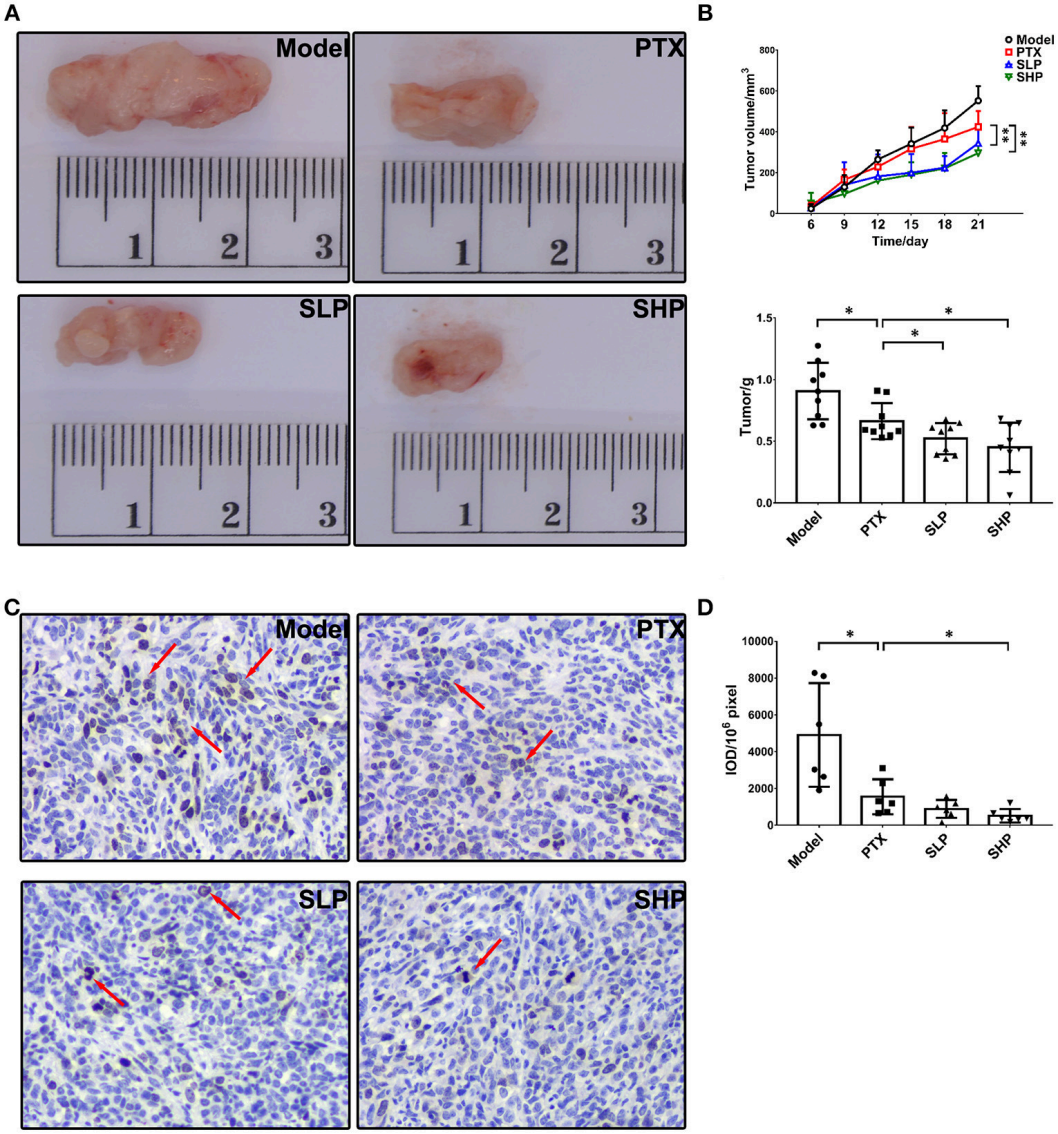
Tumor growth observation. **(A)** Tumor volume (*n* = 9). Tumor volume change between groups were compared by repeat measurement ANOVA. **(B)** Tumor mass (*n* = 9). **(C,D)** Immunohistochemistry for ki67 in tumor and the representative image (200×, *n* = 6). The mean density of positive area was calculated as ratio of integrated optical density to the total pixel of each picture (IOD/10^6^ pixel). Values were represented the means ± SD. *p < 0.05 and **p < 0.01.

In the preliminary experiment *in vitro*, SGP showed no improvement on the cytotoxicity of PTX, in which SGP was up to 200 μg/mL and PTX was 243.2 ng/mL (IC_50_), and the treatment time was 48 and 72 h (Table S2). Therefore, the enhancement possibility of SGP on PTX was studied in the murine 4T1-breast cancer model. Compared with PTX group, SHP, and SLP groups displayed improved tumor control in the murine breast cancer model (Figures 1A,B). Tumors of Model group kept growing throughout the 21-day observation, resulting in a weight of 0.907 ± 0.229 g. PTX alone inhibited the tumor growth since 15th day, resulting in a decreased tumor weight (0.663 ± 0.146 g, *p* < 0.05). By contrast, PTX plus SGP showed an earlier suppression on tumor growth, and curves of tumor volume change of SLP (200 mg/kg) and SHP (400 mg/kg) groups were significantly different from that of PTX group (both *p* < 0.01). Finally, tumors from SLP and SHP groups were 0.521 ± 0.127 g (*p* < 0.05) and 0.451 ± 0.200 g (*p* < 0.05), evidently smaller than that of PTX group. With the data of our previous data, the combination index (CI) of SGP (400 and 200 mg/kg) and PTX (12.5 mg/kg) were 1.29 and 1.12 (>0.85), indicating that there would be an additive effect or synergy in the combination of SGP and PTX against breast cancer (Table S2). Moreover, expression of ki67, the symbol protein for proliferation, was even more down-regulated in SHP group than that in PTX group (Figures 1C,D, *p* < 0.05).

### SGP Supplement Boosted Tumor Immune Surveillance by Suppressing Immune Checkpoints

**Figure 2:**
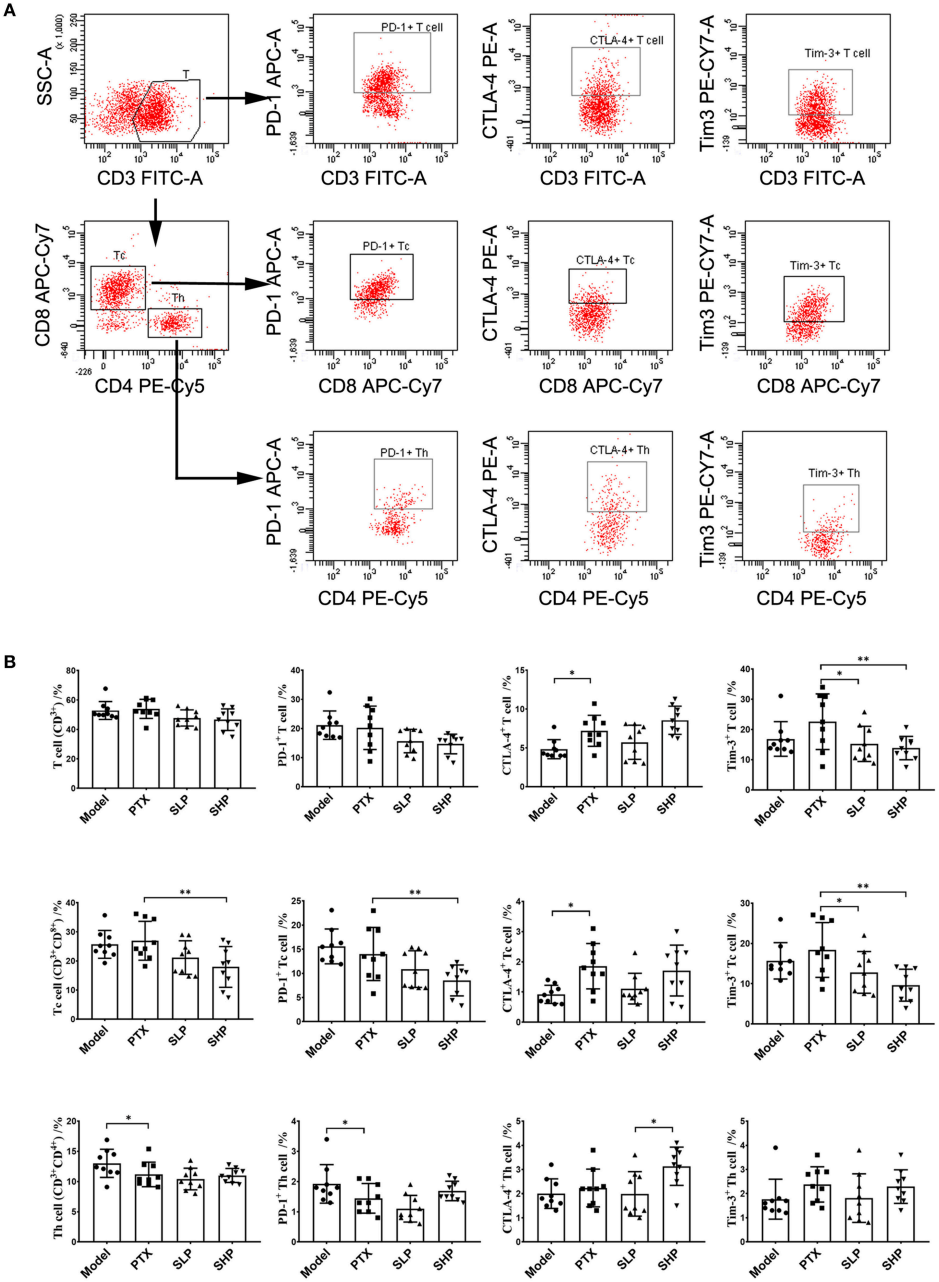
Tumor infiltrating lymphocyte (TIL) analysis by flowcytometry. **(A)** Flowcytometry analysis scheme presented by dotplot. **(B)** Proprotion of TIL subsets (first line panel) and the immune checkpoint-positive TILs (second to fourth line panels) comparison. Values were represented the means ± SD (*n* = 9). *p < 0.05 and **p < 0.01.

To explore the underlying mechanism of the anti-cancer promotion of SGP, we firstly took a look of its effect on TILs. The flowcytometry analysis scheme was presented in Figure 2A. Total T cells within TME were not apparently affected by PTX or the combination treatment, although percentage of Tc cells showed a decline in SHP group when compared with PTX group (Figure 2B). However, immune checkpoints in the TILs were obviously influenced. For the total T cells (Figure 2B, first row), it was in SHP group and SLP group that the proportions of Tim-3-positve ones were reduced, and that of PD-1-positive was tend to be decreased; while in PTX group, proportions of T cell that expressed CTLA-4 (*p* < 0.05) and Tim-3 (*p* = 0.069) were increased. For Tc cells (Figure 2B, second row), SHP and SLP groups displayed significant reductions in cells that were PD-1- and Tim-3-possitive when compared with those from PTX group. However, PD-1 and Tim-3 expressions in the helper T cells (Th) seemed to be not affected by the combination treatment groups, while the CTLA-4 expression was up-regulated (Figure 2B, third row).

### PTX Plus SGP Inhibited Tumor Metabolism Within Tumor Tissue

**Figure 3:**
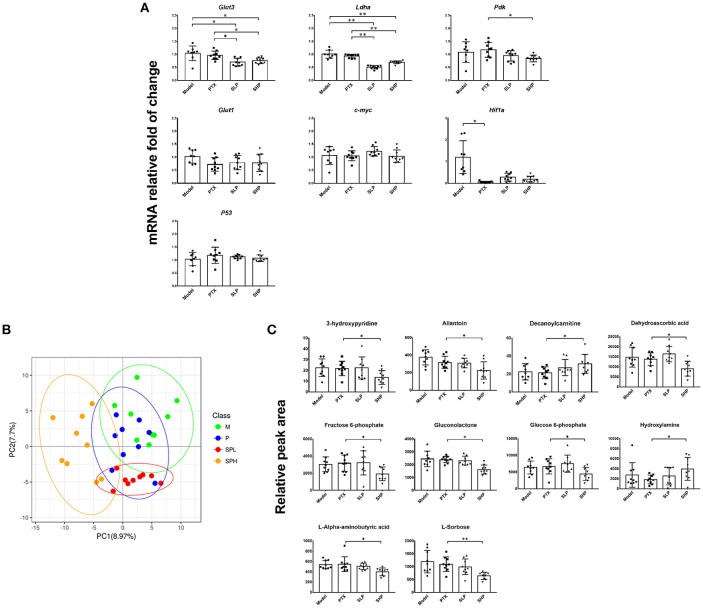
Metabolomics analysis. **(A)** q-PCR analysis for mRNA of Warburg effect-related proteins. **(B)** PLS-DA analysis for metabolite profile. **(C)** Differential metabolites comparison. Values were represented the means ± SD (*n* = 9). *p < 0.05 and **p < 0.01.

Firstly, it was found that the combination treatment exhibited significant inhibition on mRNA of several Warburg effect-related proteins (Figure 3A). In contrast to the mild suppressing tendency in PTX group, evident down-regulation was found in SHP and SLP groups, including those on mRNA levels of glucose transporter 3 (*Glut3*), lactate dehydrogenase A (*Ldha*), and pyruvate dehydrogenase kinase (*Pdk*). However, neither treatment of SHP or SLP promoted the down-regulations of hypoxia-inducible factor 1-α (*Hif1a*) caused by PTX.

Tumor metabolism profile was analyzed by GC-TOF/MS-based metabolomics to explore the improvement potential of PTX combined with SGP. By PLS-DA, overall metabolite profile of SHP group displayed evident difference from that of Model group and PTX group (Figure 3B). Then OPLS-DA was applied to made a further confirmation, in which “Q^2^(cum) > 0” indicated a significant difference (Table 2). Results showed that metabolite profile of SHP group could be apparently discriminated from that of Model group [SHP vs. Model, Q^2^(cum) = 0.557] and that of PTX group [SHP vs. PTX, Q^2^(cum) = 0.305], while the difference between model group and PTX group was not obvious [Q^2^(cum) = −0.272, Table 1]. Based on the relative peak area, differential metabolites between SHP and PTX group were obtained using a multi-criteria assessment in the OPLS-DA model (Table S3 “OPLSA” sheets, VIP ≥ 1), and then were confirmed by the univariate statistical analysis (Table S3 “*T*-test” sheets, *p* < 0.05). Among the most affected metabolite, typical intermediates within TME (glucose 6-phosphate and fructose 6-phosphate), malignant proliferation indicators [allantoin (Ahn et al., 2014) and dehydroascorbic acid (Spielholz et al., 1997)] and other 5 metabolites (6-phosphonoglucono-D-lactone, L-alpha-aminobutyric acid, L-sorbose, gluconolactone, 3-hydroxypyridine) were significantly reduced in tumors from SHP group when compared with those from PTX group; while hydroxylamine and decanoylcarnitine were enriched in SHP group (Figure 3C), suggesting that alternation of metabolic characteristic would be contribute to the tumor-control improvement by combination of PTX and SGP.

**Table 2:** Results of OPLS-DA model parameters.

	***R*^**2**^X(cum)**	***R*^**2**^Y(cum)**	**Q^**2**^(cum)**	**RMSEE**	**Pre**	**Ort**	**pR^**2**^Y**	**pQ^**2**^**	**Yaxis**	**PermI**
PTX vs. Model	0.264	0.963	−0.272	0.109	1	2	0.956	0.789	0.013	1,000
SPL vs. Model	0.227	0.979	0.258	0.165	1	2	0.325	0.205	−0.021	1,000
SPH vs. Model	0.193	0.952	0.557	0.36	1	1	0.005	0.004	−0.216	1,000
SPL vs. PTX	0.27	0.992	−0.439	0.0521	1	2	0.4	0.936	0.062	1,000
SPH vs. PTX	0.212	0.921	0.305	0.308	1	1	0.563	0.067	−0.205	1,000
SPH vs. SPL	0.209	0.922	0.325	0.153	1	1	0.596	0.069	−0.142	1,000

### SGP Supplement Restored the Gut Dysbiosis Induced by PTX

#### Overall Structural Modulation After Treatment

**Figure 4:**
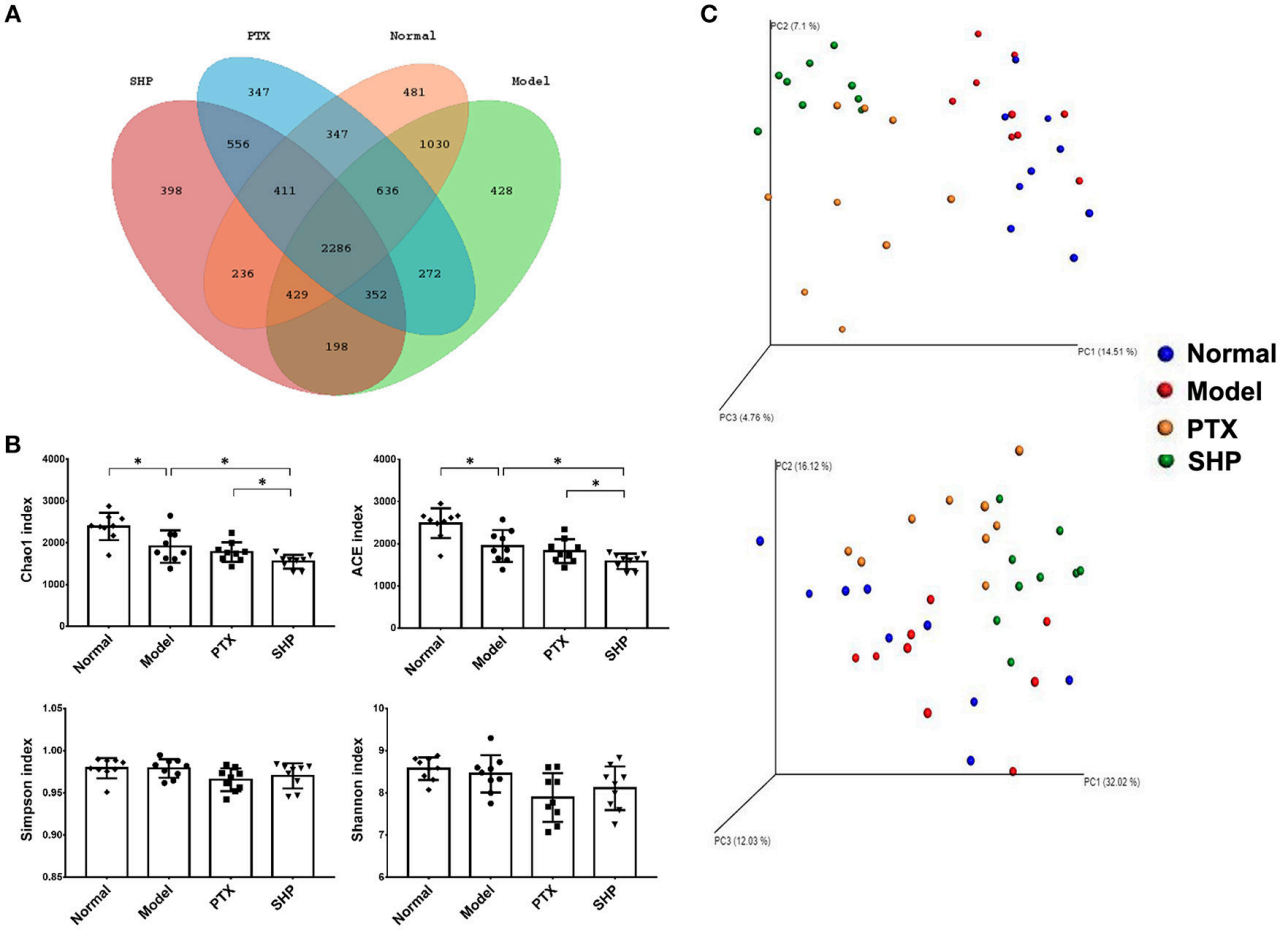
Microbiota diversity analysis. **(A)** Venn diagram showed common OTUs comparison with the four groups. **(B)** α-diversity indices. **(C)** Unweighted (upper panel) and weighted (lower panel) uniFrac PCoA assessment. Values were represented the means ± SD. *p < 0.05.

Increasing evidence suggests that gut microbiota stands the very core of therapeutic responses for tumors occurring outside of the intestinal tract (Gopalakrishnan et al., 2018; Routy et al., 2018b). Hence, we investigated its possible involvement in the efficacy of SGP in this part. Gut microbiota in caecum content from the Normal group, Model group, PTX group, and SHP group were compared by 16sRNA sequencing since the above data indicated that SHP group exhibited better tumor control, more evident suppression on TIL immunocheckpoints and tumor metabolism. Common OTU analysis presented by Venn diagram indicated that there existed 481 unique OTUs in Normal group, 428 in Model group, 347 in PTX group, and 3,398 in SPH group, respectively, while 2,286 common OTUs were shared by all samples (Figure 4A).

α-diversity analysis was applied to evaluated richness and diversity of microbiota community. Briefly, community richness could be indicated by Chao1 and ACE indices, while community diversity and uniformity was represented by Shannon, and Simpson indices. Compared with that of Normal group, obvious reduction in richness was demonstrated by lowered Chao1 and ACE indices in the gut microbiome of Model group, PTX group, and SPH group (*p* < 0.05, Figure 4B). In particular, microbiome from SPH group displayed an ever lower richness than that of Model group.

Overall community structure was compared by β-analysis using PCoA. Both unweighted and weighted PCoA assessment showed that, despite the decrease of richness, microbiome structure of Model group shared similar pattern with that of Normal group, while neither samples of PTX group or SHP group displayed alike community structure when compared to Normal group (Figure 4C). Additionally, ANOSIM made a further confirmation on the structure change induced by PTX plus SGP (Table 3). In brief, unweighted and weighted R statistic of Normal-Model comparison were 0.2229 (*p* = 0.009) and −0.0233 (*p* = 0.553), suggesting an obvious structure similarity between them. By contrast, R statistics for the comparison of SHP group with any other groups were much higher with extremely low *p-*value, indicating a significant inter-group difference among them.

**Table 3:** Result of the Analysis of similarities (ANOSIM).

**Group**	**Unweighted UniFrac**		**Weighted UniFrac**	
	**R statistic**	***p*-value**	**R statistic**	***p*-value**
Normal vs. Model	0.2239	0.009	−0.0233	0.553
Normal vs. SHP	0.9616	0.001	0.5813	0.001
Normal vs. PTX	0.678	0.001	0.2884	0.007
Model vs. SHP	0.9153	0.001	0.4791	0.001
Model vs. PTX	0.6197	0.001	0.3148	0.001
PTX vs. SHP	0.3594	0.001	0.3789	0.001

#### Community Membership Shifts After Treatment

**Figure 5:**
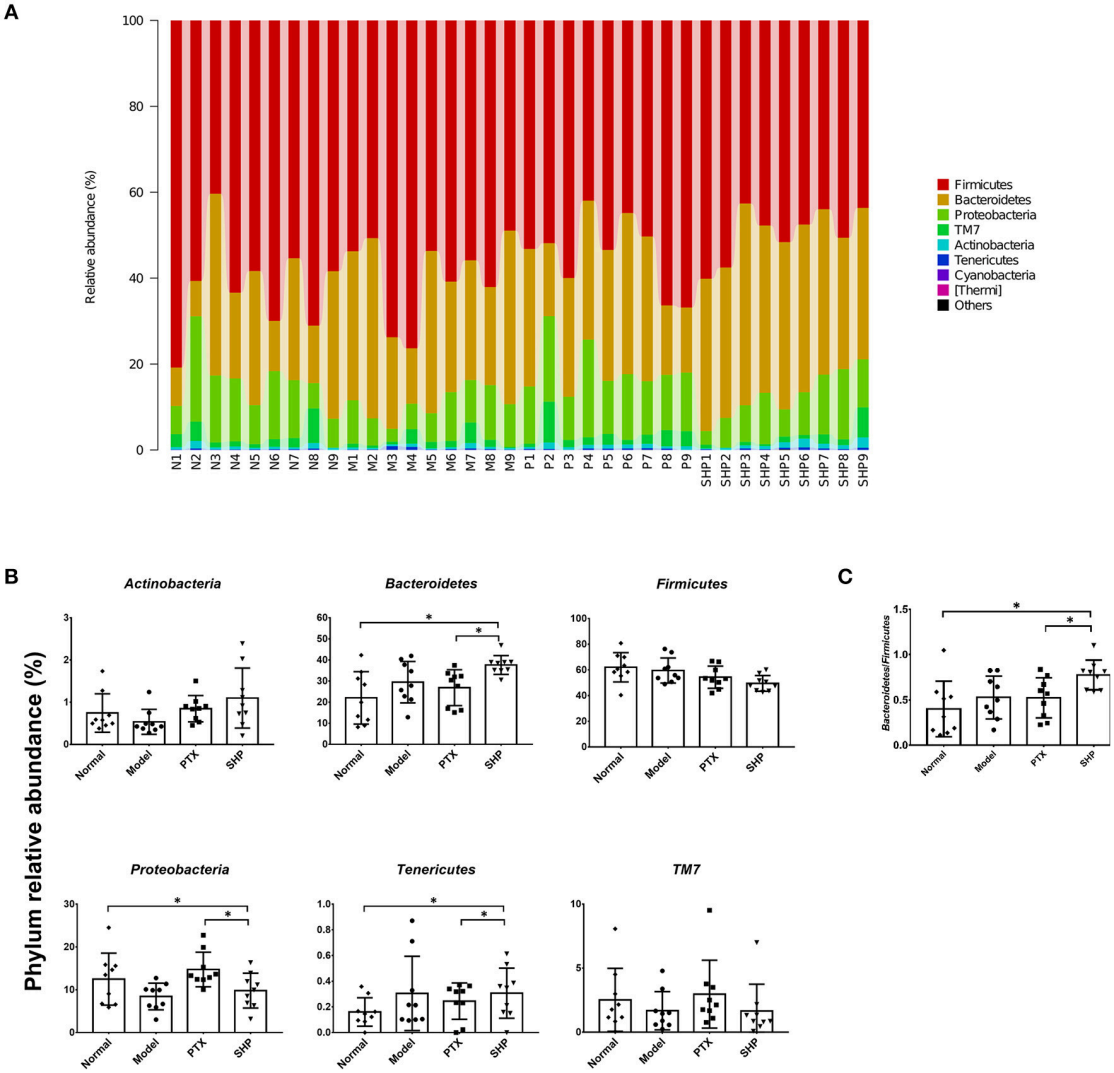
Taxonomy analysis of microbiota in the cecum content at phylum level. **(A)** Identified phyla in each sample. **(B)** Phylum relative abundance comparison (0.1%). **(C)** Ratio of *Bacteroidetes* to *Firmicutes*. Values were represented the means ± SD. *p < 0.05.

**Figure 6:**
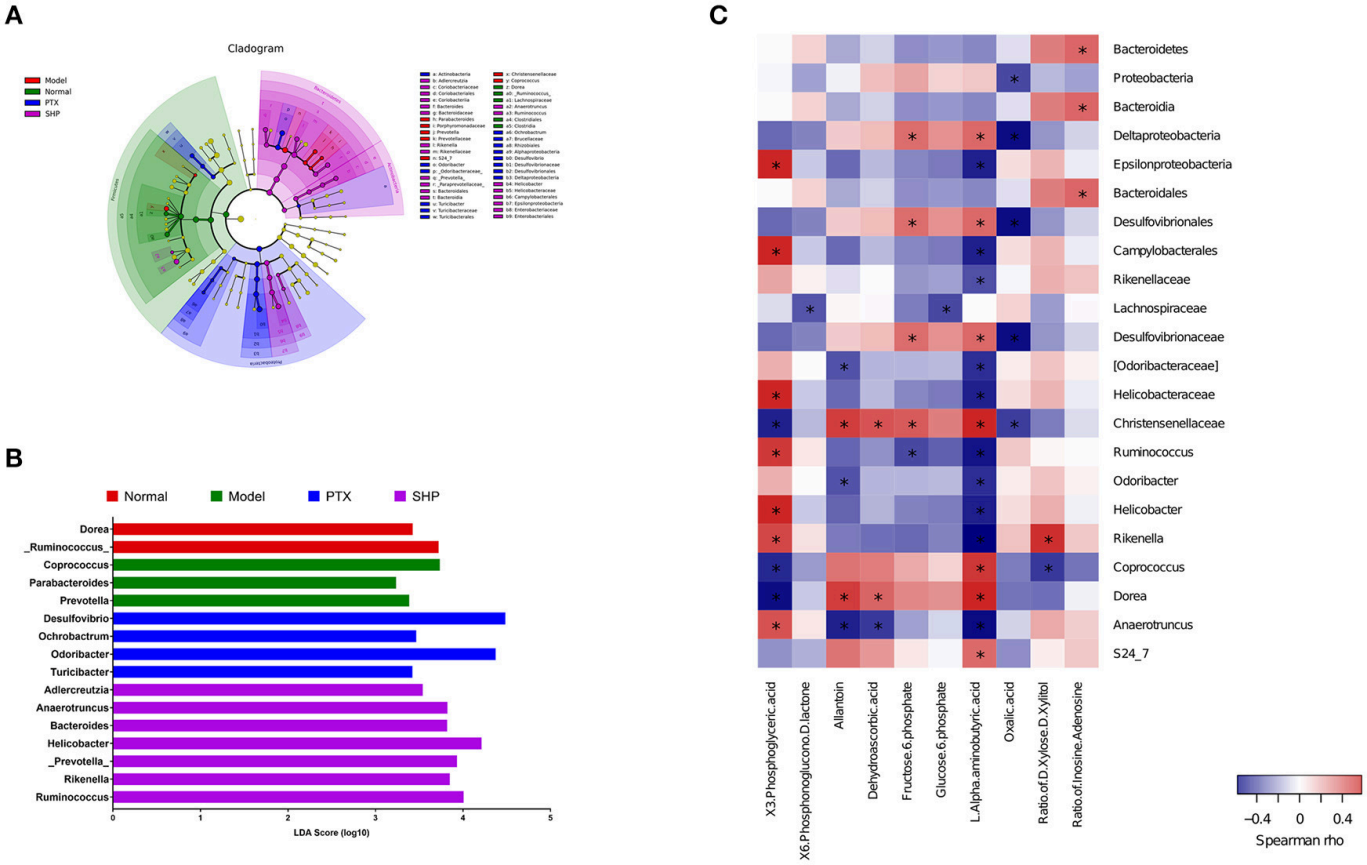
Lefse analysis and metabolic pathway enrichment analysis for microbiota in the cecum content. **(A)** Overall exhibition of Lefse analysis by cladogram. **(B)** LDA scores results of the specific enriched genera in each group. **(C)** Spearman analysis of microbiota-metabolite relationship presented by heatmap. *p < 0.05.

Taxonomy analysis revealed marked differences at both phylum and genus levels among Normal, Model, PTX and SHP groups. Overall, a total of 8 phyla were shared by samples from all groups (Figure 5A). Of them, *Firmicutes, Bacteroidetes*, and *Proteobacteria* compromised over 90% of the total classified sequences. Relative abundances (>0.1%) of *Bacteroidetes, Proteobacteria*, and *Tenericutes* displayed significant differences in the four groups. It is noteworthy that *Bacteroidetes* was evidently increased in SHP group, while that of *Firmicutes* was not apparently affected, resulting in an increased ratio of *Bacteroidetes* to *Firmicutes* (Figures 5B,C). At genus level, a total of 70 genera were identified from all samples (Table S4). LEfSe analysis indicated that there existed several specific genera in each group (Figures 6A,B). In brief, relative abundances of *Dorea* and *Ruminococcus* were highest in Normal group. *Coprococcus, Parabacteroides*, and *Prevotella* were enriched in Model group. *Desulfovibrio, Ochrobactrum, Odoribacter*, and *Turicibacter* were specific in PTX group. *Bacteroides, Ruminococcus*, and other 5 genus were significantly enriched in SHP group. Moreover, based on the datasets of metabolites (Table S3) and bacterial genus (Table S4), spearman correlation analysis showed that content of fructose-6-phosphate within tumor was negatively related to the enrichment of *Ruminococcus* (Figure 6C, *p* < 0.05).

### Microbiome Function Regulation After Treatment

**Figure 7:**
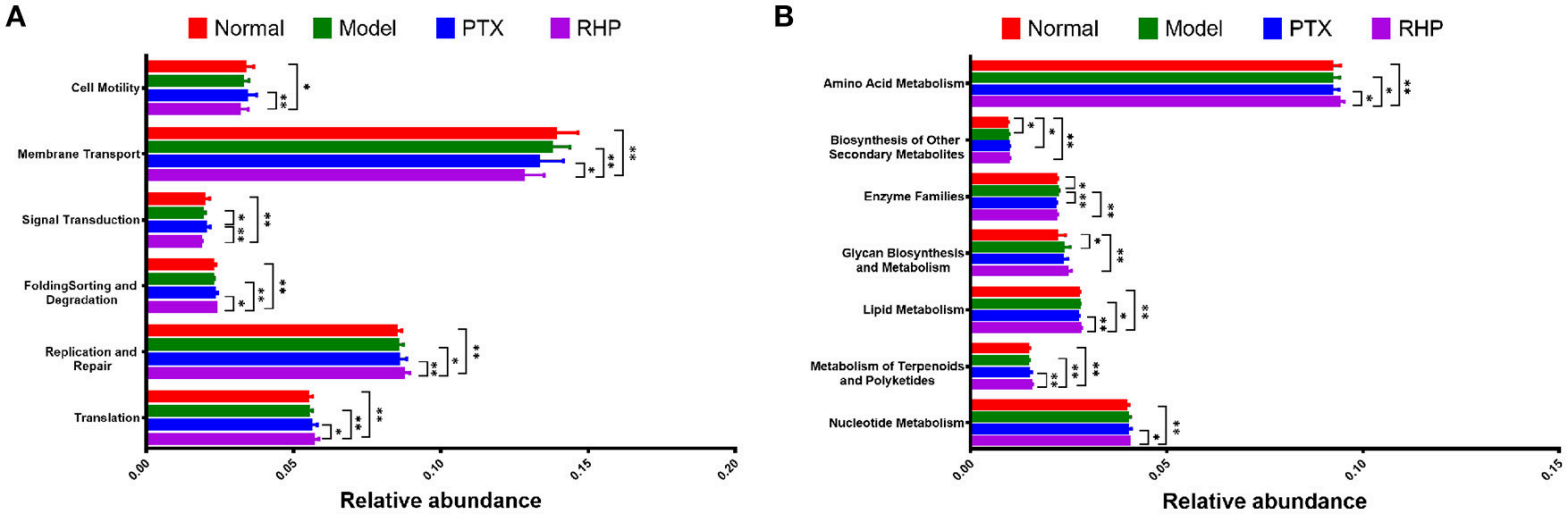
Metabolic pathway enrichment analysis. The predicted genes and their functions were aligned to KEGG database, and the relative expressions for each pathway were compared. **(A)** The most affected pathway for cellular processes (cell motility), environmental information processing (membrane transport, signal transduction), and genetic information processing (folding sorting and degradation, replication and repair, and translation). **(B)** The most affected pathway for metabolism. Values were represented the means ± SD. *p < 0.05 and **p < 0.01.

Via comparing the sequencing data with those collected in KEGG pathway database by PICRUSt, gene profile responsible for function pathways demonstrated significant differences among the four groups, including 1 for cellular processes, 2 for environmental information processing, 3 for genetic information processing, and 7 for metabolism pathways (Figure 7, Table S5). Particularly, microbiota from the tumor-bearing mice of PTX group showed significant up-regulation on pathways of cell motility and signal transduction; while that from SHP group displayed evident suppression on these two pathways, and genes responsible for the metabolism of terpenoids and polyketides were enriched.

## Discussion

PTX is a broad-spectrum natural anti-cancer reagent. Nevertheless, drug resistance and adverse effects call for elevation on its efficacy and safety (Gupta et al., 2014; Starobova and Vetter, 2017). It was previously demonstrated that SGP alone is able to potentiate the Tc-based tumor immune surveillance with a benefit reshaping on gut microbiota (Su et al., 2018). As has been found that enhanced responses to cancer therapy require an intact and optimal commensal microbiota (Viaud et al., 2013; Sivan et al., 2015), we took a further insight into the promotion on the anti-cancer activity of PTX by SGP.

In the murine breast cancer model, combination of PTX and SGP displayed an improved tumor control, with an earlier suppression of tumor growth and evident inhibition on ki67 expression than PTX alone. Under tumor development, TILs face a hostile environment that induces exhaustion to impair their antitumor activity (Boldajipour et al., 2016; Beckermann et al., 2017), which is characterized by the up-regulation of immune checkpoints, such as CTLA-4, PD-1, and Tim-3 (Ahmadzadeh et al., 2009). Results showed that combination of PTX and SGP restored the exhausted antitumorigenic immune cells (especially Tc) via inhibiting the expressions of immune checkpoints (PD-1 and Tim3), while PTX alone even evidently increased that of CTLA-4, suggesting a recovered tumor immune surveillance. As previous evidence has proved that SGP is incapable of direct lymphocyte stimulation or cytotoxicity (Su et al., 2018), the present data suggested that PTX supplementing with SGP would bring about a beneficial alternation within TME, thus improving the cell sensitivity to PTX, meanwhile enhancing the antitumorigenic immune.

Metabolism features within TME not only fuels the rapid proliferation of tumor cells, but also pose suppression on the antitumor T cells. For tumor cells, they employ spontaneous aerobic glycolysis (the Warburg effect) to intake large amount of glucose and other nutrients (lipids, amino acids), not only for energy production (adenosine triphosphate, ATP), but also more importantly, for the molecular building blocks required for cell proliferation (Ward and Thompson, 2012). Critical metabolic transporters and enzymes, like GLUTs, LDHA, and PDK, are responsible for the Warburg effect (Warburg, 1956; Ward and Thompson, 2012). Not surprisingly, oncogenic transcription factor, such as p53, HIF-1α, c-Myc, involved in malignant transformation is directly linked to the altered tumor metabolism. For example, HIF-1α induced by hypoxia is able to enhance glucose transport by increasing expression of glucose transporters 1–3 along with the transcription of pyruvate dehydrogenase kinase (Guillaumond et al., 2013). Increased c-Myc elicits numerous metabolic effects through reprogrammed gene expression, including GLUTs, LDHA (Boroughs and DeBerardinis, 2015). Nevertheless, p53 blocks excessive entry of glucose through glycolytic flux by inhibiting expression of GLUT1, GLUT3, GLUT4, phosphoglycerate mutase 1 (PGM 1) (Kruiswijk et al., 2015). As showed in our data, combination of PTX and SGP made evident down-regulations on mRNAs of *Glut3, Ldha*, and *Pdk*, while PTX alone did not affect them, indicating a possible suppression on glucose uptake. Meanwhile, obvious changes on the tumor metabolic profiling was found under the combination treatment, of which the accumulation of typical intermediates within TME was inhibited, such as glucose 6-phosphate, fructose 6-phosphate, and 6-phosphonoglucono-D-lactone, and several malignant proliferation indicators, such as allantoin (Hammad et al., 2011; Ahn et al., 2014) and dehydroascorbic acid (Spielholz et al., 1997), were also decreased. On the other hand, lactic acid in tumors of none of the treated groups (PTX, SLP, or SHP group) showed difference from that of Model group. We speculate that the effect of combination on tumor metabolism was so slow that downstream of it (such as lactic acid) had not yet been exhausted during the experiment, so the relative concentration of it was not different among the four groups. The underlying details will be explored in the further study of SGP. All the above data suggested that PTX plus SGP would inhibit the tumor metabolism within TME and induce dramatic metabolic profile alternation, so as to improve the cell sensitivity to PTX in addition to the microtubule polymerization by PTX (Peng et al., 2014).

Besides the alternation of tumor metabolism profile, combination of PTX and SGP demonstrated a restoration on the gut dysbiosis induced by PTX, which not only remodeld the microbiota community, but also regulated the microbiome function. α-diversity data showed that Chao1 and ACE indices of SHP group was lower than those of Model group and PTX group, indicating that the combination treatment decreased community richness. β-diverstiy analysis displayed significant inter-group difference between SHP group and any other group, suggesting that the combination posed apparent structure shift on the overall microbiome community. As α-diversity and β-diverstiy analysis indicated that combination of PTX and SGP endowed the tumor-bearing mice with unique gut microbiota characteristics, taxonomy analysis was employed to figure out the responsible species. Certain bacterium has been proved to be benefit to the outcomes of chemotherapies or immunotherapies (Iida et al., 2013; Viaud et al., 2013; Gopalakrishnan et al., 2018; Routy et al., 2018b). For instance, the anticancer efficacy of CTLA-4 mono-antibody relies on the existence of *Bacteroides fragilis*, which is associated with the maturation and function-gaining of dentritic cells, as well as the induction of an MHC class II-restricted Th1 cell memory response (Vétizou et al., 2015; Routy et al., 2018a). The abundance of *Ruminococcus* is negative related to the incidence of colorectal cancer (Borges-Canha et al., 2015; Wang et al., 2017). *Helicobacter* and *Rikenella* were positively correlated to enhanced immune response (Huang et al., 2017). As showed by the data, it was the combination treatment that, at phylum level, significantly raised the ratio of *Bacteroidetes* to *Firmicutes* by enriching the Gram-negative *Bacteroidetes* but not affecting the Gram-positive *Firmicutes*. At genus level analyzed by LEfSe, it was found that *Bacteroides, Ruminococcus*, and other 5 genus were specifically enriched in the tumor-bearing mice under the combination treatment, while the two cancer-risk genera, *Desulfovibrio* and *Odoribacter*, were suppressed. And it is quite interesting that abundance of *Ruminococcus* was significantly negative-associated with the amount of frucotose-6-phosphate within the tumor, an important intermediate of tumor metabolism. Additionally, several microbiome function pathways displayed distinct regulations upon the combination treatment. It is noteworthy that complex polysaccharide is one of the major driving forces in shaping the function profile of the gut microbiota (El Kaoutari et al., 2013; Tamura et al., 2017). As has been proved in our previous work, SGP is mainly made up of 50% polysaccharide, and it displayed a regulation on the microbiome function (Su et al., 2018). So it is probable that by oral supplement, SGP was able to directly reshape the gut microbiome homeostasis through regulating its metabolism mode, and serves as selective promoters to certain species. Hence, the combination treatment reduced the community richness (with reduced Chao1 and ACE indexes) but not affected diversity, with an apparent community membership shift. All these data suggested that PTX supplemented with SGP was capable of restoring the gut dysbiosis induced by PTX monotherapy, which also contributed to the suppression of tumor metabolism, resulting in an improved tumor control.

Colletively, data from the present study revealed an adjuvant candidate of SGP with PTX. The combination of PTX and SGP was not only capable of suppressing the tumor metabolism within TME, but also made a benefit reshaping on the gut microbiota. Moreover, the combination also recovered the impaired TILs via down-regulating the co-inhibitory signaling (PD-1 and/or CTLA-4). Hence in all probability, the combination of PTX and SGP against breast cancer relies on the regulation of tumor metabolism and gut microbiota, highlighting important clinical implications for the application of SGP.

## Author Contributions

JS, YX, and XZ conceived and designed the experiments. JS, DLi, ML, LS, TL, DLiang, GL, OS, and CJ performed the experiments. JS, DLi, QC, and QW analyzed the data. JS, DLi, YX, and XZ drafted and revised the manuscript.

### Conflict of Interest Statement

DLi, ML, DLiang, GL, OS, CJ, and YX were employed by Guangdong Yuewei Edible Fungi Technology Co. Ltd. The remaining authors declare that the research was conducted in the absence of any commercial or financial relationships that could be construed as a potential conflict of interest.
