# Corrigendum: Anti-breast Cancer Enhancement of a Polysaccharide From Spore of *Ganoderma lucidum* With Paclitaxel: Suppression on Tumor Metabolism With Gut Microbiota Reshaping

**DOI:** 10.3389/fmicb.2019.01224

**Published:** 2019-05-31

**Authors:** Jiyan Su, Dan Li, Qianjun Chen, Muxia Li, Lu Su, Ting Luo, Danling Liang, Guoxiao Lai, Ou Shuai, Chunwei Jiao, Qingping Wu, Yizhen Xie, Xinxin Zhou

**Affiliations:** ^1^State Key Laboratory of Applied Microbiology Southern China, Guangdong Provincial Key Laboratory of Microbial Culture Collection and Application, Guangdong Institute of Microbiology, Guangzhou, China; ^2^School of Pharmaceutical Science, Guangzhou University of Chinese Medicine, Guangzhou, China; ^3^Guangdong Yuewei Edible Fungi Technology Co. Ltd., Guangzhou, China; ^4^Department of Breast Disease, Guangdong Provincial Hospital of Chinese Medicine, Guangzhou University of Chinese Medicine, Guangzhou, China; ^5^School of Pharmacy and Chemistry, Dali University, Dali, China; ^6^Guangdong Laboratory Animals Monitoring Institute, Guangzhou, China; ^7^School of Pharmacy, Guangxi University of Chinese Medicine, Xining, China

**Keywords:** spore of *Ganoderma lucidum*, paclitaxel, tumor metabolism, immune checkpoints, gut microbiota

In the original article, there was a mistake in Figure 2 as published. In Figure 2B, the second and third histograms in the last row were the same. The third histogram should be the result of the “CTLA-4^+^ Th cell,” rather than that of the “PD-1^+^ Th cell.” The corrected Figure 2 appears below.

**Figure 2:**
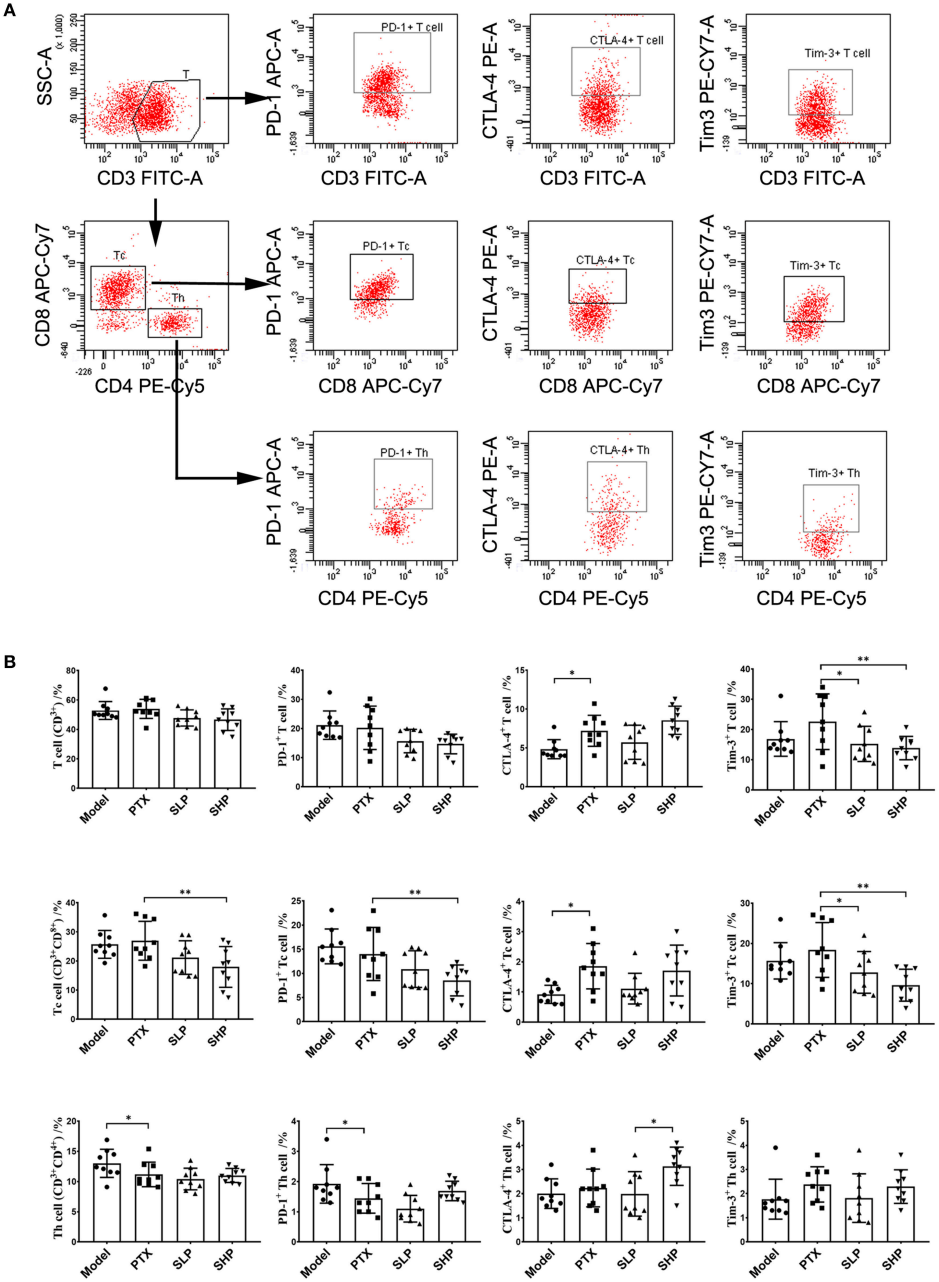
Tumor infiltrating lymphocyte (TIL) analysis by flowcytometry. **(A)** Flowcytometry analysis scheme presented by dotplot. **(B)** Proprotion of TIL subsets (first line panel) and the immune checkpoint-positive TILs (second to fourth line panels) comparison. Values were represented the means ± SD (*n* = 9). ^*^p < 0.05 and ^**^p < 0.01.

The authors apologize for this error and state that this does not change the scientific conclusions of the article in any way. The original article has been updated.

