# Supplementary material for: Anti-breast Cancer Enhancement of a Polysaccharide From Spore of Ganoderma lucidum With Paclitaxel: Suppression on Tumor Metabolism With Gut Microbiota Reshaping
Source: Front Microbiol. 2018 Dec 17;9:3099. doi: 10.3389/fmicb.2018.03099 (PMC6304348; doi:10.3389/fmicb.2018.03099)
Supplement: Supplementary file 1 [file Table_1.DOCX]

**Supplement materials 1**

**Sample Preparation and Gas Chromatograph–Time-of-Flight MS (GC-TOF/MS) Analysis for Metabolomics**

About 100mg tumor samples were homogenized with cold 50% methanol in ice-bath and centrifuged for 5 min (4 ℃, 3, 000 g) to separate debris or a lipid layer. Each sample aliquot of 50 μL was mixed with 10 μL of internal standard, and subjected to protein precipitation with 175 μL of pre-chilled methanol/chloroform (v/v=3/1). The mixture was vortexed for 30 s. After freezed at −20 °C for 20 min, the samples were centrifuged (14, 000 g, 4 ℃, 20 min). 200 μL supernatant was carefully transferred to an autosampler vial, evaporated briefly to remove chloroform, and lyophilized. Next, the lyophilized sample was derivatized with 50 μL of methoxyamine (20 mg/mL in pyridine) at 30 ℃ for 2 hr, followed by the incubation with 50 μL of N-methyl-N- (trimethylsilyl)trifluoroacetamide (1% trimethylchlorosilane) containing fatty acid methyl ester (C7-C30, FAMEs) as retention indices at 37.5 ℃ for another 1 hr. After vortexed for 1 min and kept at room temperature for 1 h, 1 μL aliquot of the sample was detected in a time-of-flight mass spectrometry (GC-TOF/MS) system (Pegasus HT, Leco Corp., St. Joseph, MO, USA) with an Agilent 7890B gas chromatography and a Gerstel multipurpose sample MPS2 with dual heads (Gerstel, Muehlheim, Germany). A Rxi-5 ms capillary column (Restek corporation, Bellefonte, PA, USA) was used for separation. The detailed instrument settings are briefly described in Table S 1- 1. Instrument optimization was performed every 24 hours. A comprehensive set of rigorous quality control (QC)/ assurance procedures is employed to ensure a consistently high quality of analytical results, throughout controlling every single step from sample receipt at laboratory to final deliverables.

Table S 1- 1 GC-TOF/MS setting

| GC | |
| --- | --- |
| Column | Rxi-5MS (Crossbond * 5% diphenyl / 95% dimethyl polysiloxane); 30 m × 250μm I.D., 0.25 μm film thickness |
| Oven Programmed Temp. (℃) | 80 (2 min), 80-300 (12 ℃/min), 300 (4.5 min), 300-320 (40 ℃/min), 320 (1 min) |
| Inlet Temp. (℃) | 230 |
| Injection Vol. (μl) | 1.0 (splitless) |
| Carrier Gas | Helium (99.9999%) |
| Transfer Interface Temp. (℃) | 270 |
| Flow Rate (mL/min) | 1.0 |
| MASS SPECTROMETER | |
| Ionization Mode | Electron impact |
| Dlectron Energy (EV) | -70 |
| Detector Voltage (v) | -1450 |
| Source Temp. (℃) | 220 |
| Acquisition Rate | 25 spectra/sec |
| Mass Range (Da) | 50-250 |

The raw data generated by GC-TOF/MS were processed using XploreMET for automated baseline denoting and smoothing, peak picking and deconvultion, creating reference database from the pooled QC samples, metabolite signal alignment, missing value correction and imputation, and QC correction. Metabolite annotation was performed by comparing the retention indices and mass spectral data with those previously generated from reference standards of known structures with XploreMET.
