# Supplementary material for: Anti-breast Cancer Enhancement of a Polysaccharide From Spore of Ganoderma lucidum With Paclitaxel: Suppression on Tumor Metabolism With Gut Microbiota Reshaping
Source: Front Microbiol. 2018 Dec 17;9:3099. doi: 10.3389/fmicb.2018.03099 (PMC6304348; doi:10.3389/fmicb.2018.03099)
Supplement: Supplementary file 2 [file Table_2.DOCX]

**Supplement materials 2**

1. **Interaction of SGP and paclitaxel (PTX) *in vitro***
   1. **Results**

Murine metastatic breast cancer 4T1 cell line was obtained from Cell bank of Chinese Academy of Sciences, Shanghai, China. 4T1 cells were cultured in high glucose DMEM medium (4.5 mg/mL, Gibco, NY, USA) supplemented with 10% fetal bovine serum (FBS, Gibco, NY, USA) and 1% penicillin/streptomycin (Gibco, NY, USA), and maintained in humidified incubators at 37 °C under an atmosphere of 5% CO2.

4T1 cells were seeded in a 96-well plate at a density of 1.25 x 10^4^ cells/mL (sextuple wells in each group) in complete DMEM medium. Firstly, the seeded 4T1 cells were treated with multiple concentrations of PTX (15.6, 31.25, 62.5, 125, 250, 500, 1000, 2000, and 4000 ng/mL) for 48h and 72h. Then the medium was replaced with 100 μL of complete DMEM medium containing 0.5 mg/mL 3-4, 5-dimethyl- 2-thiazolyl)-2,5-diphenyl-2-H-tetrazolium bromide (MTT) for another 4-hour incubation. At last, the medium was discarded and 150 μL DMSO was add to dissolve the formazan. The optical density was measured at 490 nm on a microplate reader to obtain the inhibition rate. The half maximal inhibitory concentration (IC_50_) of PTX was calculated with SPSS 22 (IBM Corp., NY, USA).

To analyze the interaction of SGP and PTX, 4T1 cells were simultaneously treated with PTX (at the IC_50_ of 48h) and SGP (1.56, 3.13, 6.25, 12.5, 25, 50, 100, and 200 μg/mL) for 48h and 72h. The inhibition rate was calculated as mentioned before.

- 1. **Results**

IC_50_ of PTX at 48h and 72h were 243.2 ng/mL and 151.9 ng/mL, respectively. However, the inhibition of PTX (243.2 ng/mL) on 4T1 was not evidently promoted by the supplement of SGP (Figure S 1), suggesting that there is not any additive or synergic effect between SGP and PTX *in vitro*.

Figure S 1 Interaction of SGP and paclitaxel (PTX) *in vitro*. Values were represented the means ± SD (n = 6).

1. **Combination index calculation**
   1. **Methods**

Firstly, tumor inhibition rate (IR) was calculated as follows: IR = (1-Weight_treatment_/Weight_model_) × 100%, where Weight means the average tumor mass. To make the synergy assessment, the combination index (CI) was calculated as described by Wang *et al* (1) with data from our previous study. CI =IR_com_/[IR_PTX_+(1-IR_PTX_) ×IR_SGP_], in which IR_com_ was the tumor growth inhibition rate of combination group (SHP and SLP group), IR_PTX_ was that of PTX mono-treatment group, and IR_SGP_ was that of SGP mono-treatment groups from our published data (2). CI = 0.85~1.15 indicated an additive effect, CI > 1.25 indicated a synergy, and CI < 0.85 indicated an antagonism.

- 1. **Results**

Results showed that CI of SGP (400mg/kg and 200mg/kg) and PTX (12.5mg/kg) were 1.29 and 1.12 (>0.85), indicating that there would be an additive effect or synergy in the combination of SGP and PTX against breast cancer (Table S 2- 1).

Table S 2- 1 Combination index calculation

| Group | | Tumor mass (mg, mean±SD) | IR ^a^ (%) | CI ^b^ |
| --- | --- | --- | --- | --- |
| Mono-treatment study | Model | 517±127 | / | / |
|  | SGPL (EL) | 387±66 | 12.1 | / |
|  | SGPH (EH) | 450±116 | 23.4 | / |
| Combination study | Model | 907±229 | / | / |
|  | PTX | 663±146 | 26.9 | / |
|  | SPL | 521±127 | 42.6 | 1.12 |
|  | SPH | 451±200 | 50.3 | 1.29 |

a: tumor inhibition rate (IR) was calculated as follows: IR = (1-Weight_treatment_/Weight_model_) × 100%, where Weight means the average tumor mass.

b: CI =IR_com_/[IR_PTX_+(1-IR_PTX_) ×IR_SGP_], in which IR_com_ was the tumor growth inhibition rate of combination group (SHP and SLP group), IR_PTX_ was that of PTX mono-treatment group, and IR_SGP_ was that of SGP mono-treatment groups.

Reference

1. Wang, Y., Wang, Z.W., Huang, Z.S., Wu, Y.N., Xu, Z.D., Synergistic and side-effect attenuating effects of aloe polysaccharide on several chemotherapy reagents. *Traditional Chinese Drug Research & Clinical Pharmacology* (2002) (02): 89-91.

2. Su, J., Su, L., Li, D., Shuai, O., Zhang, Y., Liang, H., et al., Antitumor Activity of Extract From the Sporoderm-Breaking Spore of Ganoderma lucidum: Restoration on Exhausted Cytotoxic T Cell With Gut Microbiota Remodeling. *Frontiers in Immunology* (2018) 9 (1765). 10.3389/fimmu.2018.01765
